# Supplementary material for: Dynamic transcriptomic profiles of zebrafish gills in response to zinc supplementation
Source: BMC Genomics. 2010 Oct 11;11:553. doi: 10.1186/1471-2164-11-553 (PMC3091702; doi:10.1186/1471-2164-11-553)
Supplement: Additional file 2 — Interactive Direct Interaction Network representing the molecular interactions between zinc, copper, iron, calcium and proteins encoded by transcripts changed by zinc supplementation. Mini web-site containing index.html and hyperlinked pages in subdirectory describing a Direct Interaction Network automatically generated based on curated interactions contained within the proprietary PathwayArchitect database. Ovals represent proteins and the circles symbolize metal ions. Objects are coloured by their abundance in zebrafish at the time-point they were significantly different from the control is a scale from -4 fold (dark green) to +4 fold (dark red). Where significant differences were found at more than one time-point, the colour overlay shows expression at the first instance. Dark blue squares denote 'binding', and light blue squares 'expression'; green squares stand for 'regulation', green diamonds for 'metabolism', and green circles for 'promoter binding'. Arrow heads indicate directionality of the interaction where annotated. All nodes and edges can be further interrogated by selecting the relative area of the image. [file 1471-2164-11-553-S2.zip › PathwayArchitect Zn xs DIN/653786.html]

# REGULATION:

|  |  |
| --- | --- |
| Type | REGULATION |
| Effect | Positive |


---

|  |  |
| --- | --- |
| Score | 0 |


---

|  |  |
| --- | --- |
| Reference Count | 3 |


---

|  |  |
| --- | --- |
| Mechanism | Unknown |


---

|  |  |
| --- | --- |
| Reference:0 || Sentence | "In the rostral medial preoptic area, Fos-ir was induced by mounts alone primarily in cells coexpressing ERalpha-ir, while Fos-ir was induced by intromissions mainly in cells coexpressing both ERalpha-ir and ERbeta-ir (ERalpha/ERbeta-ir)." |
| PMID | 12788290 |
| Year | 2003 |
| Species | Rat |
| Journal | Horm Behav |
| RefScore | 1 |
| Source | PArchNLP |
  |
|  |  |


---

|  |  |
| --- | --- |
 Reference:1 || Sentence | "Administration of the chemicals to ERalpha knockout mice did not activate IGF-IR, indicating that ERalpha is required for activation of uterine IGF-IR by these diverse chemicals." |
| PMID | 10965916 |
| Year | 2000 |
| Species | Mouse |
| Journal | Endocrinology |
| RefScore | 2 |
| Source | PArchNLP |
  ||  |  |


---

|  |  |
| --- | --- |
 Reference:2 || Sentence | The stimulation of IGF-IR expression by Rg1 in MCF-7 cells appears to require ER, and its actions might involve ligand-independent activation of ER. |
| PMID | 16415910 |
| Year | 2006 |
| Species | Human |
| Journal | Br J Pharmacol |
| RefScore | 2 |
| Source | PArchNLP |
  |


---

|  |  |
| --- | --- |
